# Multilayer network changes in patients with migraine

**DOI:** 10.1002/brb3.3316

**Published:** 2023-11-08

**Authors:** Jinseung Kim, Dong Ah Lee, Ho‐Joon Lee, Kang Min Park

**Affiliations:** ^1^ Department of Family Medicine Busan Paik Hospital, Inje University College of Medicine Busan South Korea; ^2^ Departments of Neurology Haeundae Paik Hospital, Inje University College of Medicine Busan South Korea; ^3^ Departments of Radiology Haeundae Paik Hospital, Inje University College of Medicine Busan South Korea

**Keywords:** magnetic resonance imaging, migraine, neural network

## Abstract

**Introduction:**

To investigate changes in the multilayer network in patients with migraine compared to healthy controls.

**Methods**: This study enrolled 82 patients with newly diagnosed migraine without aura and 53 healthy controls. Brain magnetic resonance imaging (MRI) was conducted using a 3‐tesla MRI scanner, including three‐dimensional T1‐weighted and diffusion tensor imaging (DTI). A gray matter layer matrix was created with a morphometric similarity network using T1‐weighted imaging and the FreeSurfer program. A white matter layer matrix was also created with structural connectivity using the DTI studio (DSI) program. A multilayer network analysis was then performed by applying graph theory using the BRAPH program.

**Results:**

Significant changes were observed in the multilayer network at the global level in patients with migraines compared to the healthy controls. The multilayer modularity (0.177 vs. 0.160, *p* = .0005) and average multiplex participation (0.934 vs. 0.924, *p* = .002) were higher in patients with migraines than in the healthy controls. In contrast, the average multilayer clustering coefficient (0.406 vs. 0.461, *p* = .0005), average overlapping strength (56.061 vs. 61.676, *p* = .0005), and average weighted multiplex participation (0.847 vs. 0.878, *p* = .0005) were lower in patients with migraine than in the healthy controls. In addition, several regions showed significant changes in the multilayer network at the nodal level, including multiplex participation, multilayer clustering coefficients, overlapping strengths, and weighted multiplex participation.

**Conclusion:**

This study demonstrated significant changes in the multilayer network in patients with migraines compared to healthy controls. This could aid an understanding of the complex brain network in patients with migraine and may be associated with the pathophysiology of migraines. Patients with migraine show multilayer network changes in widespreading brain regions compared to healthy controls, and specific brain areas seem to play a hub role for pathophysiology of the migraine.

## Graphical abstract

- Multilayer network analysis treats each network as a layer and enhances dimensionality by considering the interplay among various brain networks.
- This study employed multilayer network analysis to examine alterations in the gray and white matter layers in migraine.
- Significant changes were observed in the multilayer network at the global level and nodal level in patients with migraines.

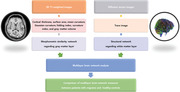

## 1 INTRODUCTION

Migraine is characterized by throbbing headaches that are aggravated by physical activity. It is a chronic neurological disorder accompanied by photophobia, phonophobia, nausea, and vomiting alongside headaches (“Headache Classification Committee of the International Headache Society (IHS) The International Classification of Headache Disorders, 3rd edition,” 2018; Kim et al., 2012). Migraines are not only very common, with a prevalence of up to 17% in our country, but also rank second among all diseases in terms of disability in daily life according to the Global Burden of Disease study published in 2017 (Group, 2017; Kim et al., 2012; Kim et al., 2013). In particular, the number of disability‐adjusted‐life years is highest among people between the ages of 15 and 49 years who are socially active; this reduces the quality of life of patients and imposes a heavy socioeconomic burden (Steiner et al., 2018).

The exact pathophysiology of migraines has not yet been elucidated. The once‐popular vascular theory of migraines, which proposes that blood vessel dilatation causes migraines and that the aura of migraines is caused by vasoconstriction, is no longer considered plausible. Vasodilatation, if it occurs during spontaneous migraine attacks, is likely an epiphenomenon caused by an unstable central neurovascular control mechanism (Charles, 2009). Currently, the pathophysiology of migraine is thought to be that neuronal dysfunction leads to a series of intracranial and extracranial changes responsible for migraine, including the four phases of premonitory symptoms, aura, headache, and postdrome (Cutrer, 2006). In addition, cortical spreading depression, trigeminovascular system activation, sensitization, serotonin, and calcitonin gene‐related peptides can be related to migraine pathophysiology (Charles & Pozo‐Rosich, 2019; Kaube et al., 2002; Panconesi, 2008; Paolucci et al., 2021; Sarchielli et al., 2001).

Furthermore, in recent studies using resting‐state functional magnetic resonance imaging (rs‐fMRI) and structural magnetic resonance imaging (MRI), various brain network changes have been reported in patients with migraine, such as disruptions of the pontine, thalamic, and sensorimotor and visual networks (Messina et al., 2022; Park et al., 2022). Most recently, a multilayer network analysis method has been introduced in neuroscience and applied to various neurological diseases; this has improved the understanding of neurological disorders (Canal‐Garcia et al., 2022; Guillon et al., 2017; Huang et al., 2020; Lv et al., 2021). This method does not analyze multiple brain networks individually but rather analyzes each network as a layer and increases the dimension by considering the relationship between multiple brain networks (Lv et al., 2021). It consists of multiple layers or interconnected networks, each representing a distinct relationship or interaction between nodes. As a representative method, functional connectivity based on rs‐fMRI and structural connectivity based on diffusion tensor imaging (DTI) can be analyzed simultaneously by increasing the dimension, and structural and functional networks can be considered together (Huang et al., 2020). It can also be analyzed by considering the relationship between amyloid‐positron emission tomography and structural MRI (Canal‐Garcia et al., 2022), or by considering multifrequency bands in electroencephalography (Guillon et al., 2017).

However, no study has utilized multilayer network analysis in patients with migraines. Therefore, this study investigated changes in the multilayer network in patients with migraine compared to healthy controls. Multilayer network changes in the gray and white matter layers were investigated using a morphometric similarity network and structural connectivity, respectively. The authors hypothesized that there would be a clear difference in the multilayer network between patients with migraines and healthy controls.

## 2 METHODS

### 2.1 Participants: patients with migraine and healthy controls

This study was conducted at a single tertiary headache center and was approved by the institutional review board of the hospital. Patients with migraine without aura were enrolled from August 2018 to December 2022 according to the following criteria: (1) newly diagnosed patients with migraine without aura according to the International Classification of Headache Disorders, 3rd edition (“Headache Classification Committee of the International Headache Society (IHS) The International Classification of Headache Disorders, 3rd edition,”^2018^) and (2) brain MRI, including three‐dimensional T1‐weighted imaging and DTI, at the time of diagnosis. The exclusion criteria were as follows: (1) structural lesions on brain MRI, (2) poor‐quality T1‐weighted or diffusion tensor images for analysis, or (3) medical or neurological disorders other than migraines. The medical records were reviewed, and demographic and clinical characteristics such as age, sex, age at headache onset, disease duration (time between the age at headache onset and MRI), headache attack frequency per month, presence of aura, and headache intensity on a visual analog scale were investigated.

Age‐ and sex‐matched healthy participants with no medical or neurological disorders, such as migraine, were enrolled as a control group. All patients had normal brain MRI scans without any structural lesions.

### 2.2 MRI acquisition: T1‐weighted images and diffusion tensor images

Patients with migraines and healthy controls underwent brain MRI with the same sequences using the same 3‐Tesla MRI scanner equipped with a 32‐channel head coil (AchievaTx, Phillips Healthcare). All brain MRI scans were performed in the interictal state without headache in patients with migraines. The MR sequences were routine MRI protocols for patients with migraine in the study center, including three‐dimensional fluid‐attenuated inversion recovery, coronal T2‐weighted imaging, three‐dimensional T1‐weighted imaging, and DTI. Three‐dimensional T1‐weighted images were obtained using a turbo‐field echo sequence with the following parameters: TI = 1300 ms, repetition time/echo time (TR/TE) = 8.6/3.96 ms, flip angle (FA) = 8°, and 1 mm^3^ isotropic voxel size. The specific DTI parameters were as follows: 32 different diffusion directions, *b*‐values of 0 and 1000 s/mm^2^ (*b*0 images were acquired once), TR/TE = 8620/85 ms, FA = 90°, slice thickness = 2.25 mm, acquisition matrix = 120 × 120, field of view = 240 × 240 mm^2^, and parallel imaging factor (SENSE) = 2.

### 2.3 Multilayer network analysis

**FIGURE 1:**
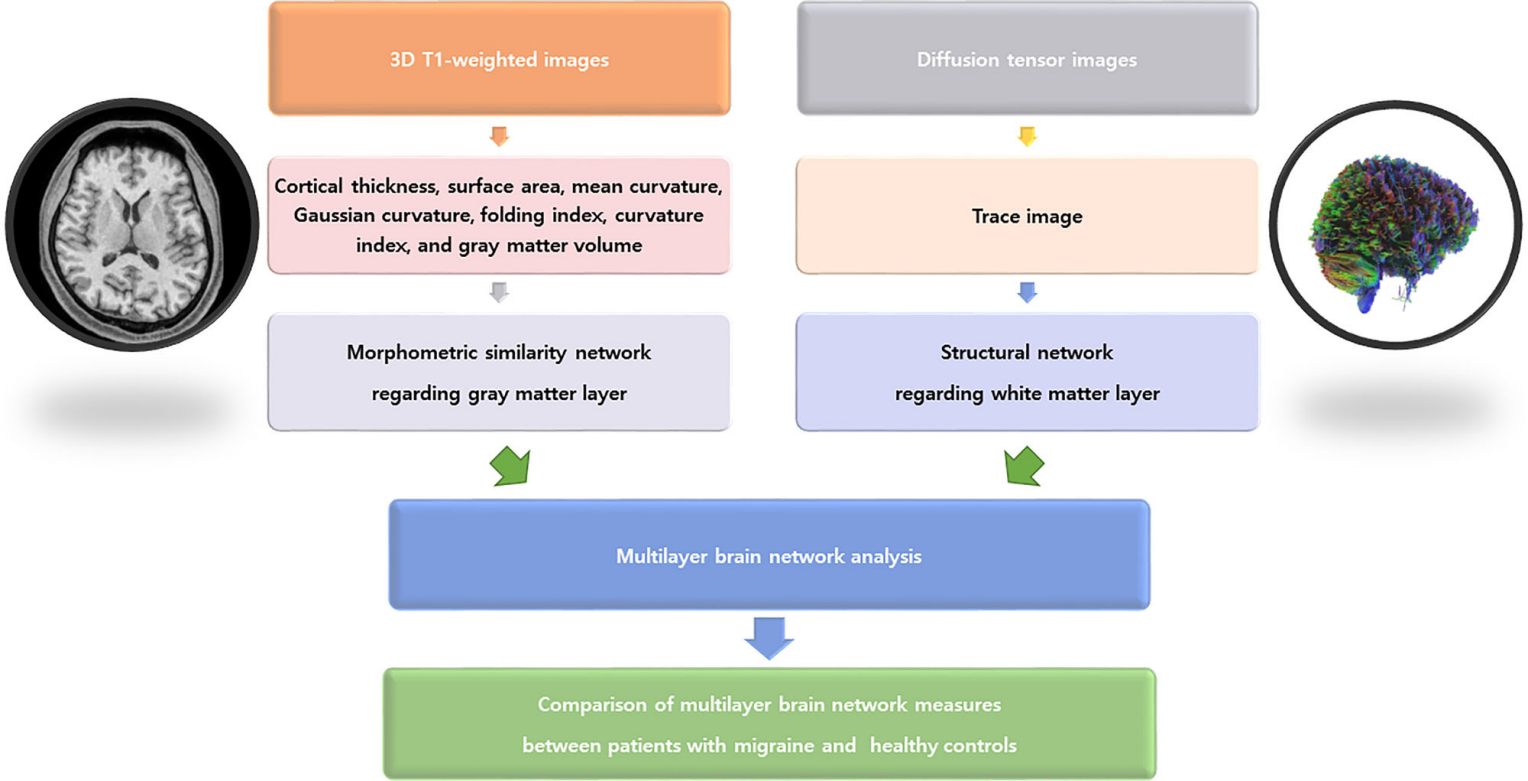
Multilayer network analysis process. A gray matter layer matrix with a morphometric similarity network was created using three‐dimensional T1‐weighted imaging and the FreeSurfer program, and a white matter layer matrix with structural connectivity was created using the diffusion tensor imaging studio program. A multilayer network analysis was then performed by applying graph theory using the BRAPH program.

Figure 1 shows the multilayer network analysis process in this study. First, a gray matter layer matrix was created with a morphometric similarity network using T1‐weighted images. Using the FreeSurfer program (version 7.0) and the “recon‐all” processing stream with default parameters, multiple morphometric features, including cortical thickness, surface area, mean curvature, Gaussian curvature, folding index, curvature index, and gray matter volume (Fischl & Dale, 2000), were obtained for the 62 regions of interests (ROIs) in each hemisphere as defined by the Desikan‐Killiany atlas (Desikan et al., 2006). The morphometric similarity between each pair of ROIs was estimated using Pearson's correlation between their morphometric features to produce a 62 × 62 matrix. This matrix was the morphometric similarity network regarding the gray matter layer (King & Wood, 2020; Seidlitz et al., 2018).

Second, a white matter layer matrix was created with structural connectivity from the DTI. A DTI studio (DSI) program (version 2021) was used to preprocess DTI, which included open‐source images, correcting the eddy current and phase distortion artifacts, setting up a mask (thresholding, smoothing, and defragments), and reconstruction with a generalized Q‐sampling imaging method (Yeh et al., 2010). Then, fiber tracking was conducted using a deterministic method, and a 62 × 62 matrix was produced as defined by the Desikan‐Killiany atlas; this was a connectivity analysis of the white matter layer. Third, a multilayer network analysis was performed using a graph theory using a BRAPH program (version 2.0) (Mijalkov et al., 2017). A “pipeline connectivity multiplex comparison WU,” which was the pipeline script to compare two groups of participants using connectivity matrix data and weighted graphs, was selected. The Desikan‐Killiany atlas and two connectivity matrices made in the previous steps were loaded, and connectivity multiplex analyses were performed based on weighted graphs corrected for age and sex.

Multilayer network measures, such as the (i) average degree of overlap, (ii) average flexibility, (iii) multilayer modularity, (iv) average multilayer clustering coefficient, (v) average multiplex participation, (vi) average overlapping degree, (vii) average overlapping strength, (viii) persistence, and (ix) average weighted multiplex participation, were calculated at the global level (Buldu & Papo, 2018; Casas‐Roma et al., 2022; De Domenico, 2018; Lv et al., 2021; Puxeddu et al., 2021; Shahabi et al., 2023). Additionally, multilayer network measures, including the (i) multilayer clustering coefficient, (ii) multiplex participation, (iii) overlapping strength, and (iv) weighted multiplex participation, were calculated at the node level.

The average degree of overlap was defined as the average number of edges connected to a node in all the layers. The flexibility of each node was calculated as the number of times it changed the community assignment normalized by the total possible number of changes. Multilayer modularity was defined as the multilayer quality function of the resulting partition in a multilayer network. The average multilayer clustering coefficient was the average of the two multiplex clustering coefficients in all nodes. It was defined as the ratio of the number of triangles in the graph to the number of connected triples of nodes. The average overlapping strength was the sum of the strengths of a node in all layers. Persistence was calculated as the normalized sum of the number of nodes that did not change their community assignments. Average multiplex participation was defined as the average homogeneity of the number of neighbors across layers (Buldu & Papo, 2018; Casas‐Roma et al., 2022; De Domenico, 2018; Lv et al., 2021; Puxeddu et al., 2021; Shahabi et al., 2023). The differences in these network measures were compared between patients with migraine and the healthy controls.

### 2.4 Statistical analysis

The Student's *t*‐test was used for age comparisons, and Fisher's exact test was used to compare sex between the groups. All statistical tests were performed using MedCalc® (MedCalc Software version 20.014; Ostend, Belgium; https://www.medcalc.org; 2021). Nonparametric permutation tests with 1000 permutations were used to compare the network measures, which were executed directly within the BRAPH program. Additionally, *p* values < .05 were considered statistically significant. Using Bonferroni corrections, a *p*‐value threshold with multiple corrections was applied to analyze network measure comparisons between groups. Thus, because the seven network measures were compared between the two groups at the global level, it was deduced that only *p* < .0071 (0.05/9 = 0.0055) was significant. At the nodal level, because 62 nodes were compared in both groups, only *p* < .0008 (0.05/62 = 0.0008) was considered statistically significant.

## 3 RESULTS

### 3.1 Clinical characteristics of participants

Table 1 shows the clinical characteristics of the patients with migraine and healthy controls. Age and sex did not differ between the patients with migraines and healthy controls (age: 30.6 vs. 29.2 years, *p* = .334; males: 15/82 (18.3%) vs. 9/53 (17.0%), *p* = .846).

**TABLE 1:** Clinical characteristics in patients with migraine.

	Patients with migraine (*N* = 82)	Healthy controls (*N* = 53)	*p* Value
Age, years (± standard deviation)	30.6 (± 10.2)	29.2 (± 4.4)	.334
Male, *n* (%)	15 (18.3)	9 (17.0)	.846
Age of headache onset, years (interquartile range)	20 (12–30)		
Disease duration, months (interquartile range)	60 (24–120)		
Attack frequency per month, *n* (interquartile range)	4 (1–8)		
Presence of aura, *n* (%)	0 (0)		
Headache intensity, visual analog scale (interquartile range)	7 (6–8)		

### 3.2 Multilayer network analysis

#### 3.2.1 Network measures at the global level in the multilayer network analysis

Significant changes were observed in the multilayer network at the global level in the patients with migraines compared to the healthy controls (Table 2). The multilayer modularity (0.177 vs. 0.160, *p* = .0005) and average multiplex participation (0.934 vs. 0.924, *p* = .002) were higher in patients with migraine than in the healthy controls. In contrast, the average multilayer clustering coefficient (0.406 vs. 0.461, *p* = .0005), average overlapping strength (56.061 vs. 61.676, *p* = .0005), and average weighted multiplex participation (0.847 vs. 0.878, *p* = .0005) were lower in patients with migraine than in the healthy controls. However, the average degree of overlap, flexibility, overlapping degree, and persistence did not differ between the groups.

**TABLE 2:** The differences of the network measures at the global level in a multilayer network analysis between the patients with migraine and healthy controls.

	Patients with migraine	Healthy controls	Differences	Lower value of the 95% confidence interval	Upper value of the 95% confidence interval	*p* Value
Average degree overlap	24.183	25.932	1.749	−1.462	1.545	.022
Average flexibility	0.535	0.555	0.020	−0.038	0.036	.284
Multilayer modularity	0.177	0.160	−0.017	−0.011	0.011	*.001
Average multilayer clustering coefficient	0.407	0.462	0.055	−0.021	0.022	*.001
Average multiplex participation	0.934	0.924	−0.011	−0.007	0.007	*.003
Average overlapping degree	76.435	79.636	3.201	−2.482	2.542	.013
Average overlapping strength	56.062	61.676	5.614	−2.352	2.529	*.001
Persistence	0.465	0.453	−0.013	−0.037	0.038	.507
Average weighted multiplex participation	0.847	0.879	0.031	−0.014	0.014	*.001

*Statistical significance.

#### 3.2.2 Network measures at the nodal level in the multilayer network analysis

Several regions exhibited significant changes in the multilayer network at the nodal level, including the multilayer clustering coefficient, overlapping strength, and weighted multiplex participation.

**FIGURE 2:**
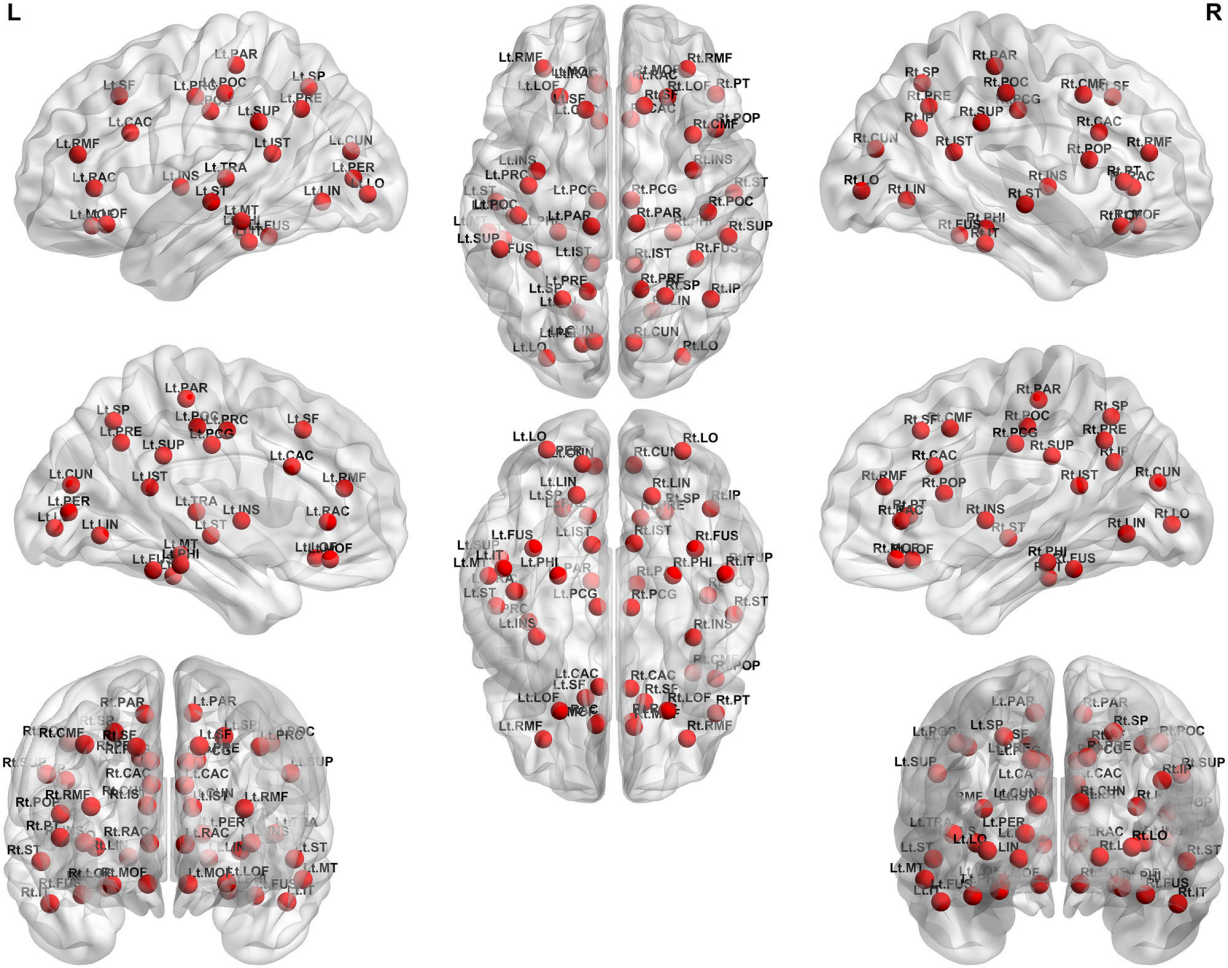
Significant differences in the multilayer clustering coefficient at the nodal level in the multilayer network analysis between the patients with migraine and healthy controls. The red nodes indicate regions showing lower multilayer clustering coefficients at the nodal level in a multilayer network analysis in patients with migraines compared with healthy controls. CAC, caudal anterior cingulate gyrus; CUN, cuneus; FUS, fusiform gyrus; IP, inferior parietal gyrus; IT, inferior temporal gyrus; IST, isthmus cingulate gyrus; LO, lateral occipital gyrus; LOF, lateral orbitofrontal gyrus; LIN, lingual gyrus; MOF, medial orbitofrontal gyrus; MT, middle temporal gyrus; PHI, parahippocampal gyrus; PAR, paracentral gyrus; PT, pars triangularis gyrus; PER, pericalcarine gyrus; POP, pars opercularis; POC, postcentral gyrus; PCG, posterior cingulate gyrus; PRC, precentral gyrus; PRE, precuneus; RAC, rostral anterior cingulate gyrus; RMF, rostral middle frontal gyrus; RMF, rostral middle frontal gyrus; SF, superior frontal gyrus; SP, superior parietal gyrus; ST, superior temporal gyrus; SUP, supramarginal gyrus; TRA, transverse temporal gyrus; INS, insula.

The multilayer clustering coefficient of the bilateral caudal anterior cingulate, bilateral cuneus, bilateral fusiform, right inferior parietal, bilateral inferior temporal, bilateral isthmus cingulate, bilateral lateral occipital, bilateral lateral orbitofrontal, bilateral lingual, bilateral medial orbitofrontal, left middle temporal, bilateral parahippocampal, bilateral paracentral, right pars triangularis, left pericalcarine, bilateral postcentral, bilateral posterior cingulate, left precentral, bilateral precuneus, bilateral rostral anterior cingulate, bilateral rostral middle frontal, bilateral superior frontal, bilateral superior parietal, bilateral superior temporal, bilateral supramarginal, left transverse temporal, right caudal middle frontal, right pars opercularis, and bilateral insula gyrus was lower in patients with migraine than in the healthy controls (Figure 2).

**FIGURE 3:**
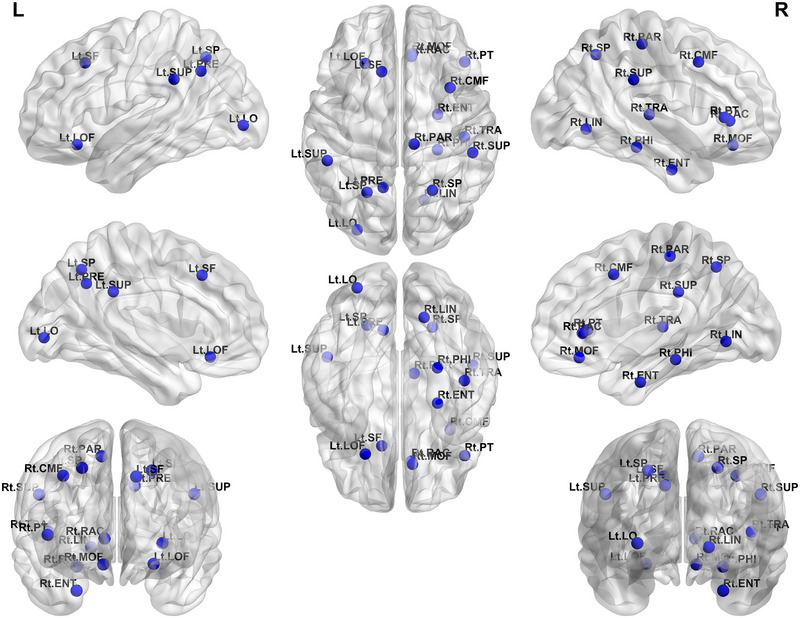
Significant differences in the overlapping strength at the nodal level in the multilayer network analysis between the patients with migraine and healthy controls. The blue nodes show the regions representing lower overlapping strength at the nodal level in a multilayer network analysis of patients with migraine compared with healthy controls. LO, lateral occipital gyrus; LOF, lateral orbitofrontal gyrus; LIN, lingual gyrus; PRE, precuneus; SF, superior frontal gyrus; SP, superior parietal gyrus; SUP, supramarginal gyrus; CMF, caudal middle frontal gyrus; ENT, entorhinal gyrus; MOF, medial orbitofrontal gyrus; PHI, parahippocampal gyrus; PAR, paracentral gyrus; PT, pars triangularis gyrus; RAC, rostral anterior cingulate gyrus; TRA, transverse temporal gyrus.

The overlapping strengths of the left lateral occipital, left lateral orbitofrontal, left precuneus, left superior frontal, bilateral superior parietal, bilateral supramarginal, right caudal middle frontal, right entorhinal, right lingual, right medial orbitofrontal, right parahippocampal, right paracentral, right pars triangularis, right rostral anterior cingulate, and right transverse temporal gyri were lower in patients with migraine than in the healthy controls (Figure 3).

**FIGURE 4:**
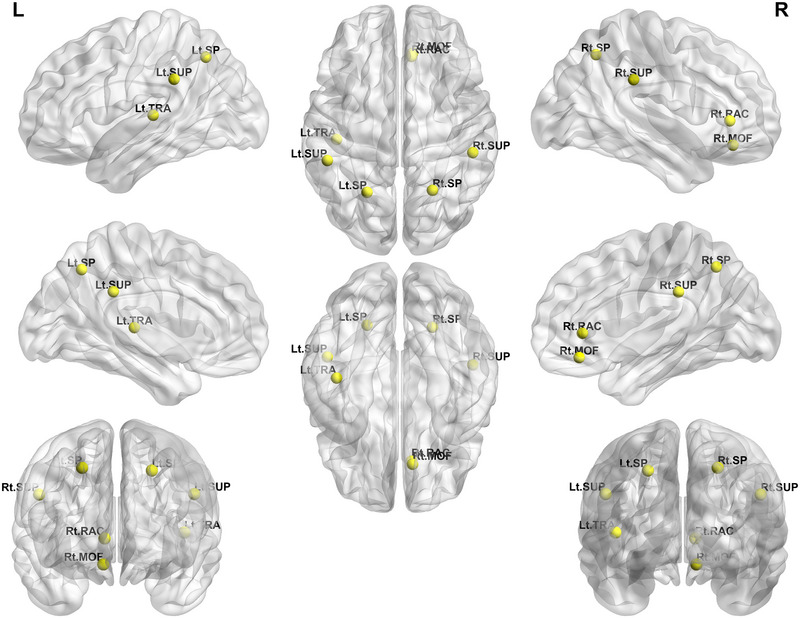
Significant differences in the weighted multiplex participation at the nodal level in the multilayer network analysis between the patients with migraine and healthy controls. The yellow nodes indicate regions showing lower weighted multiplex participation at the nodal level in a multilayer network analysis in patients with migraines compared with healthy controls. SP, superior parietal gyrus; SUP, supramarginal gyrus; TRA, transverse temporal gyrus; MOF, medial orbitofrontal gyrus; RAC, rostral anterior cingulate gyrus.

The weighted multiplex participation of the bilateral superior parietal, bilateral supramarginal, left transverse temporal, right medial orbitofrontal, and right rostral anterior cingulate gyri was lower in patients with migraine than in the healthy controls (Figure 4). However, no regions showed differences in multiplex participation between patients with migraine and the healthy controls.

## 4 DISCUSSION

This is the first study to investigate changes in the multilayer network in patients with migraine compared to healthy controls. Significant changes were observed in the multilayer network at the global level in patients with migraines. Multilayer modularity and average multiplex participation were higher in patients with migraines than in the healthy controls. In contrast, compared with the healthy controls, the average multilayer clustering coefficient, average overlapping strength, and average weighted multiplex participation were lower in patients with migraines. Additionally, several regions showed significant changes in the multilayer network at the nodal level in patients with migraines, including multiplex participation, multilayer clustering coefficients, overlapping strengths, and weighted multiplex participation.

At the global level, multilayer modularity and average multiplex participation were higher in patients with migraines than in healthy controls. Modularity quantifies the ratio of connection densities within and between communities. High modularity indicates a robust community structure in which nodes within the same community are densely connected, whereas the connections between communities are sparse. Multilayer modularity methods extend the traditional modularity measures to multilayer networks. Multilayer modularity aims to divide nodes into communities or modules that maximize the modularity score while considering the interactions between different layers (Buldu & Papo, 2018; Casas‐Roma et al., 2022; De Domenico, 2018; Lv et al., 2021; Puxeddu et al., 2021; Shahabi et al., 2023). Increased multilayer modularity in patients with migraine indicates improvement or enhancement of the modularity score obtained when comparing a multilayer network to its aggregated single‐layer representation. The average multiplex participation reflects the average number of layers in which a node is present or has a connection. It quantifies the extent to which nodes are involved in multiple layers and have relationships spanning multiple types of interactions (Buldu & Papo, 2018; Casas‐Roma et al., 2022; De Domenico, 2018; Lv et al., 2021; Puxeddu et al., 2021; Shahabi et al., 2023). Therefore, an increased average multiplex participation in patients with migraines indicated that more nodes were involved in the gray and white matter layers of their brains than in those of healthy controls. In other words, the interaction between the gray and white matter layers was particularly higher in patients with migraines.

A previous study involving a unilayer network analysis also demonstrated an increase in modularity in the global structural brain network in patients with migraine compared with healthy controls (Michels et al., 2021). However, compared to healthy controls, the average multilayer clustering coefficient, average overlapping strength, and average weighted multiplex participation were lower in patients with migraines. The multilayer clustering coefficient quantifies the average clustering coefficient of a node across all layers, accounting for both intralayer and interlayer clustering (Buldu & Papo, 2018; Casas‐Roma et al., 2022; De Domenico, 2018; Lv et al., 2021; Puxeddu et al., 2021; Shahabi et al., 2023). The mean clustering coefficient indicates how well‐neighboring nodes are connected, which is related to the segregation of the network. Therefore, an overall decreased segregation of the brain network was observed in patients with migraines. The average overlapping strength quantifies the strength of the overlapping connections between nodes across different layers. It quantifies the degree to which nodes share connections across layers and provides insight into interlayer connectivity and interactions. The strength of a connection represents the weight or intensity of the interaction between nodes (Buldu & Papo, 2018; Casas‐Roma et al., 2022; De Domenico, 2018; Lv et al., 2021; Puxeddu et al., 2021; Shahabi et al., 2023). The average weighted multiplex participation is a metric utilized in multilayer network analysis to quantify the degree to which nodes participate in multiple layers while considering the strength or weight of their connections. It provides information regarding the average weighted interconnectedness and overlap of nodes across different network layers (Buldu & Papo, 2018; Casas‐Roma et al., 2022; De Domenico, 2018; Lv et al., 2021; Puxeddu et al., 2021; Shahabi et al., 2023). Therefore, from the present study, it can be implied that the connections between the overall nodes in patients with migraine were lower than those in the healthy controls. This was consistent with a previous study with structural connectivity analysis, which revealed significantly weaker interregional connectivity strength between anatomical compartments such as the frontotemporal, parietal, and visual regions in patients with migraine (Michels et al., 2021). These changes in the relationship between these gray and white matter layers may be related to the pathophysiology of migraines.

Several regions exhibited significant changes in the multilayer network at the nodal level, including multiplex participation, multilayer clustering coefficients, overlapping strengths, and weighted multiplex participation. It was found that the change in connectivity of these nodes was not limited to some lobes but spread across the frontal, temporal, parietal, and occipital lobes. Therefore, when observed through multilayer network analysis, brain network changes in patients with migraines appear throughout the brain. In a study using tract‐based spatial statistics, it was found that compared to the normal group, patients with migraine showed significant cortical thinning in very wide brain areas such as temporal, frontal, insular, postcentral, primary and associative visual areas, which was in a line with our present study (Abagnale et al., 2023). Furthermore, a previous study conducted to determine how accurately migraine with aura was diagnosed using machine learning and brain morphologic features, including cortical thickness, cortical surface area, cortical volume, cortical mean Gaussian curvature, and cortical folding index, showed a very high accuracy of 97% (Mitrovic et al., 2023). Specifically, this study revealed that the thickness of the temporal pole, lingual gyrus, and pars opercularis were the most important features for diagnosing patients with migraine. This result was consistent with our present study. In this study, we found lower multilayer clustering coefficient of the lingual gyrus and pars opercularis, and lower overlapping strength of the lingual gyrus in patients with migraine compared to healthy controls. The importance of the lingual gyrus in the pathophysiolgy of migraine is already well established. A decreased functional connectivity between the hypothalamus and lingual gyrus was found in a previous study using functional MRI, which was also associated with migraine attack frequency (Messina et al., 2022). Moreover, a study using functional magnetic resonance spectroscopic imaging revealed mitochondrial dysfunction in patients with migraine, and reported that it was associated with the occipital lobe, including the lingual gyrus (Sandor et al., 2005). Some patients with migraine may experience visual disturbances, including photophobia and strange visual phenomena, in the visual cortex during migraine attacks, often crossing over with the affected visual field, which could be related with abnormality of multilayer network in occipital lobe. Another study also found that patients with migraine showed alterations of the cortical thickness in the pars opercularis, which was the area where mutlilayer network alteration occurred in our present study (Guarnera et al., 2021). In addition, the insula and parietal cortex was commonly found to be involved in alteration of the multilayer network analysis in our present study. Migraine's central symptom, the headache, directly affects the brain's pain processing centers, such as the insula and parietal cortex. A previous study also demonstrated that patients with migraine displayed interictal changes in the topology of intrinsic connections, with greater connectivity between primary sensory cortices and the anterior insula, a region involved in representing and coordinating responses to emotional salience (Tso et al., 2015). Considering these evidence, patients with migraine show morphometric and multilayer network changes in widespreading brain regions compared to healthy controls, and specific brain areas seem to play a hub role for pathophysiology of the migraine.

Although this was the first study to conduct a multilayer network analysis in patients with migraines, it had several limitations. First, a relatively large number of patients with migraines were enrolled to improve the homogeneity of the study participants. However, a selection bias exists because this study was conducted in a single center, and it was difficult to extend the interpretation to all patients with migraine. Second, this was a cross‐sectional study. It would have been better if the brain network changes that occurred after commencing migraine treatment in patients had been observed longitudinally. A recent study using rs‐fMRI demonstrated significant functional connectivity changes between pre‐ and post‐erenumab treatment in patients with migraine, especially in pain processing regions (Schwedt et al., 2022). Despite these limitations, the findings suggest that multilayer network analysis can be widely used in the future to reveal the pathophysiology of the disease and observe changes in the brain network in patients with various headache disorders.

## 5 CONCLUSION

This study demonstrated significant changes in the multilayer network in patients with migraines compared to healthy controls. This could aid an understanding of the complex brain network in patients with migraine and may be associated with the pathophysiology of migraines. Patients with migraine show multilayer network changes in widespreading brain regions compared to healthy controls, and specific brain areas seem to play a hub role for pathophysiology of the migraine.

## AUTHOR CONTRIBUTIONS

Jinseung Kim: Conceptualization; data curation; formal analysis; writing—original draft; writing—review and editing. Dong Ah Lee: Conceptualization; data curation; writing—review and editing; writing—original draft; visualization. Ho‐Joon Lee: Conceptualization; writing—original draft; writing—review and editing; validation; software; data curation. Kang Min Park: Conceptualization; methodology; software; formal analysis; investigation; project administration; supervision; writing—review and editing; writing—original draft.

## CONFLICT OF INTEREST STATEMENT

All authors have no conflicts of interest to declare at the time of submission.

### PEER REVIEW

The peer review history for this article is available at https://publons.com/publon/10.1002/brb3.3316.

## Data Availability

The data that support the findings of this study are available from the corresponding author upon reasonable request.

## References

[brb33316-bib-0001] Abagnale, C. , Di Renzo, A. , Sebastianelli, G. , Casillo, F. , Tinelli, E. , Giuliani, G. , Tullo, M. G. , Serrao, M. , Parisi, V. , Fiorelli, M. , Caramia, F. , Schoenen, J. , Di Piero, V. , & Coppola, G. (2023). Whole brain surface‐based morphometry and tract‐based spatial statistics in migraine with aura patients: Difference between pure visual and complex auras. Frontiers in Human Neuroscience, 17, 1146302. 10.3389/fnhum.2023.1146302 37144161 PMC10151576

[brb33316-bib-0002] Buldu, J. M. , & Papo, D. (2018). Can multilayer brain networks be a real step forward?: Comment on “Network science of biological systems at different scales: A review” by M. Gosak et al. Physics of Life Reviews, 24, 153–155. 10.1016/j.plrev.2017.12.007 29290618

[brb33316-bib-0003] Canal‐Garcia, A. , Gomez‐Ruiz, E. , Mijalkov, M. , Chang, Y. W. , Volpe, G. , & Pereira, J. B. , & Alzheimer's Disease Neuroimaging, I . (2022). Multiplex connectome changes across the Alzheimer's disease spectrum using gray matter and amyloid data. Cerebral Cortex, 32(16), 3501–3515. 10.1093/cercor/bhab429 35059722 PMC9376877

[brb33316-bib-0004] Casas‐Roma, J. , Martinez‐Heras, E. , Sole‐Ribalta, A. , Solana, E. , Lopez‐Soley, E. , Vivo, F. , Diaz‐Hurtado, M. , Alba‐Arbalat, S. , Sepulveda, M. , Blanco, Y. , Saiz, A. , Borge‐Holthoefer, J. , Llufriu, S. , & Prados, F. (2022). Applying multilayer analysis to morphological, structural, and functional brain networks to identify relevant dysfunction patterns. Network Neuroscience, 6(3), 916–933. 10.1162/netn_a_00258 36605412 PMC9810367

[brb33316-bib-0005] Charles, A. (2009). Advances in the basic and clinical science of migraine. Annals of Neurology, 65(5), 491–498. 10.1002/ana.21691 19479724

[brb33316-bib-0006] Charles, A. , & Pozo‐Rosich, P. (2019). Targeting calcitonin gene‐related peptide: A new era in migraine therapy. Lancet, 394(10210), 1765–1774. 10.1016/S0140-6736(19)32504-8 31668411

[brb33316-bib-0007] Cutrer, F. M. (2006). Pathophysiology of migraine. Seminars in Neurology, 26(2), 171–180. 10.1055/s-2006-939917 16628527

[brb33316-bib-0008] De Domenico, M. (2018). Multilayer network modeling of integrated biological systems: Comment on “Network science of biological systems at different scales: A review” by Gosak et al. Physics of Life Reviews, 24, 149–152. 10.1016/j.plrev.2017.12.006 29305153

[brb33316-bib-0009] Desikan, R. S. , Segonne, F. , Fischl, B. , Quinn, B. T. , Dickerson, B. C. , Blacker, D. , Buckner, R. L. , Dale, A. M. , Maguire, R. P. , Hyman, B. T. , Albert, M. S. , & Killiany, R. J. (2006). An automated labeling system for subdividing the human cerebral cortex on MRI scans into gyral based regions of interest. Neuroimage, 31(3), 968–980. 10.1016/j.neuroimage.2006.01.021 16530430

[brb33316-bib-0010] Fischl, B. , & Dale, A. M. (2000). Measuring the thickness of the human cerebral cortex from magnetic resonance images. PNAS, 97(20), 11050–11055. 10.1073/pnas.200033797 10984517 PMC27146

[brb33316-bib-0011] Group, G. B. D. N. D. C. (2017). Global, regional, and national burden of neurological disorders during 1990–2015: A systematic analysis for the Global Burden of Disease Study 2015. Lancet Neurology, 16(11), 877–897. 10.1016/S1474-4422(17)30299-5 28931491 PMC5641502

[brb33316-bib-0012] Guarnera, A. , Bottino, F. , Napolitano, A. , Sforza, G. , Cappa, M. , Chioma, L. , Pasquini, L. , Rossi‐Espagnet, M. C. , Lucignani, G. , Figà‐Talamanca, L. , Carducci, C. , Ruscitto, C. , Valeriani, M. , Longo, D. , & Papetti, L. (2021). Early alterations of cortical thickness and gyrification in migraine without aura: A retrospective MRI study in pediatric patients. The Journal of Headache and Pain, 22(1), 79. 10.1186/s10194-021-01290-y 34294048 PMC8296718

[brb33316-bib-0013] Guillon, J. , Attal, Y. , Colliot, O. , La Corte, V. , Dubois, B. , Schwartz, D. , Chavez, M. , & De Vico Fallani, F. (2017). Loss of brain inter‐frequency hubs in Alzheimer's disease. Scientific Reports, 7(1), 10879. 10.1038/s41598-017-07846-w 28883408 PMC5589939

[brb33316-bib-0014] Headache Classification Committee of the International Headache Society (IHS) The International Classification of Headache Disorders, 3rd edition . (2018). Cephalalgia, 38(1), 1–211. 10.1177/0333102417738202 29368949

[brb33316-bib-0015] Huang, J. , Zhu, Q. , Wang, M. , Zhou, L. , Zhang, Z. , & Zhang, D. (2020). Coherent pattern in multi‐layer brain networks: Application to epilepsy identification. IEEE Journal of Biomedical and Health Informatics, 24(9), 2609–2620. 10.1109/JBHI.2019.2962519 31899443

[brb33316-bib-0016] Kaube, H. , Katsarava, Z. , Przywara, S. , Drepper, J. , Ellrich, J. , & Diener, H. C. (2002). Acute migraine headache: Possible sensitization of neurons in the spinal trigeminal nucleus? Neurology, 58(8), 1234–1238. 10.1212/wnl.58.8.1234 11971092

[brb33316-bib-0017] Kim, B. K. , Chu, M. K. , Lee, T. G. , Kim, J. M. , Chung, C. S. , & Lee, K. S. (2012). Prevalence and impact of migraine and tension‐type headache in Korea. Journal of Clinical Neurology, 8(3), 204–211. 10.3988/jcn.2012.8.3.204 23091530 PMC3469801

[brb33316-bib-0018] Kim, B. K. , Chung, Y. K. , Kim, J. M. , Lee, K. S. , & Chu, M. K. (2013). Prevalence, clinical characteristics and disability of migraine and probable migraine: A nationwide population‐based survey in Korea. Cephalalgia, 33(13), 1106–1116. 10.1177/0333102413484990 23615490

[brb33316-bib-0019] King, D. J. , & Wood, A. G. (2020). Clinically feasible brain morphometric similarity network construction approaches with restricted magnetic resonance imaging acquisitions. Network Neuroscience, 4(1), 274–291. 10.1162/netn_a_00123 32181419 PMC7069065

[brb33316-bib-0020] Lv, Y. , Huang, S. , Zhang, T. , & Gao, B. (2021). Application of multilayer network models in bioinformatics. Frontiers in Genetics, 12, 664860. 10.3389/fgene.2021.664860 33868392 PMC8044439

[brb33316-bib-0021] Messina, R. , Gollion, C. , Christensen, R. H. , & Amin, F. M. (2022). Functional MRI in migraine. Current Opinion in Neurology, 35(3), 328–335. 10.1097/WCO.0000000000001060 35674076

[brb33316-bib-0022] Messina, R. , Rocca, M. A. , Valsasina, P. , Misci, P. , & Filippi, M. (2022). Clinical correlates of hypothalamic functional changes in migraine patients. Cephalalgia, 42(4‐5), 279–290. 10.1177/03331024211046618 34644197

[brb33316-bib-0023] Michels, L. , Koirala, N. , Groppa, S. , Luechinger, R. , Gantenbein, A. R. , Sandor, P. S. , Kollias, S. , Riederer, F. , & Muthuraman, M. (2021). Structural brain network characteristics in patients with episodic and chronic migraine. The Journal of Headache and Pain, 22(1), 8. 10.1186/s10194-021-01216-8 33657996 PMC7927231

[brb33316-bib-0024] Mijalkov, M. , Kakaei, E. , Pereira, J. B. , Westman, E. , & Volpe, G. , & Alzheimer's Disease Neuroimaging, I . (2017). BRAPH: A graph theory software for the analysis of brain connectivity. PLoS ONE, 12(8), e0178798. 10.1371/journal.pone.0178798 28763447 PMC5538719

[brb33316-bib-0025] Mitrovic, K. , Petrusic, I. , Radojicic, A. , Dakovic, M. , & Savic, A. (2023). Migraine with aura detection and subtype classification using machine learning algorithms and morphometric magnetic resonance imaging data. Frontiers in Neurology, 14, 1106612. 10.3389/fneur.2023.1106612 37441607 PMC10333052

[brb33316-bib-0026] Panconesi, A. (2008). Serotonin and migraine: A reconsideration of the central theory. The Journal of Headache and Pain, 9(5), 267–276. 10.1007/s10194-008-0058-2 18668197 PMC3452194

[brb33316-bib-0027] Paolucci, M. , Altamura, C. , & Vernieri, F. (2021). The role of endothelial dysfunction in the pathophysiology and cerebrovascular effects of migraine: A narrative review. Journal of Clinical Neurology, 17(2), 164–175. 10.3988/jcn.2021.17.2.164 33835736 PMC8053543

[brb33316-bib-0028] Park, S. , Lee, D. A. , Lee, H. J. , Shin, K. J. , & Park, K. M. (2022). Brain networks in migraine with and without aura: An exploratory arterial spin labeling MRI study. Acta Neurologica Scandinavica, 145(2), 208–214. 10.1111/ane.13536 34633068

[brb33316-bib-0029] Puxeddu, M. G. , Petti, M. , & Astolfi, L. (2021). A comprehensive analysis of multilayer community detection algorithms for application to EEG‐based brain networks. Frontiers in Systems Neuroscience, 15, 624183. 10.3389/fnsys.2021.624183 33732115 PMC7956967

[brb33316-bib-0030] Sandor, P. S. , Dydak, U. , Schoenen, J. , Kollias, S. S. , Hess, K. , Boesiger, P. , & Agosti, R. M. (2005). MR‐spectroscopic imaging during visual stimulation in subgroups of migraine with aura. Cephalalgia, 25(7), 507–518. 10.1111/j.1468-2982.2005.00900.x 15955037

[brb33316-bib-0031] Sarchielli, P. , Alberti, A. , Floridi, A. , & Gallai, V. (2001). Levels of nerve growth factor in cerebrospinal fluid of chronic daily headache patients. Neurology, 57(1), 132–134. 10.1212/wnl.57.1.132 11445643

[brb33316-bib-0032] Schwedt, T. J. , Nikolova, S. , Dumkrieger, G. , Li, J. , Wu, T. , & Chong, C. D. (2022). Longitudinal changes in functional connectivity and pain‐induced brain activations in patients with migraine: A functional MRI study pre‐ and post‐ treatment with Erenumab. The Journal of Headache and Pain, 23(1), 159. 10.1186/s10194-022-01526-5 36517767 PMC9748909

[brb33316-bib-0033] Seidlitz, J. , Vasa, F. , Shinn, M. , Romero‐Garcia, R. , Whitaker, K. J. , Vertes, P. E. , Wagstyl, K. , Kirkpatrick Reardon, P. , Clasen, L. , Liu, S. , Messinger, A. , Leopold, D. A. , Fonagy, P. , Dolan, R. J. , Jones, P. B. , Goodyer, I. M. , Raznahan, A. , & Bullmore, E. T. (2018). Morphometric similarity networks detect microscale cortical organization and predict inter‐individual cognitive variation. Neuron, 97(1), 231–247. e237. 10.1016/j.neuron.2017.11.039 29276055 PMC5763517

[brb33316-bib-0034] Shahabi, H. , Nair, D. R. , & Leahy, R. M. (2023). Multilayer brain networks can identify the epileptogenic zone and seizure dynamics. Elife, 12, 10.7554/eLife.68531 PMC10065796 36929752

[brb33316-bib-0035] Steiner, T. J. , Stovner, L. J. , Vos, T. , Jensen, R. , & Katsarava, Z. (2018). Migraine is first cause of disability in under 50s: Will health politicians now take notice? The Journal of Headache and Pain, 19(1), 17. 10.1186/s10194-018-0846-2 29468450 PMC5821623

[brb33316-bib-0036] Tso, A. R. , Trujillo, A. , Guo, C. C. , Goadsby, P. J. , & Seeley, W. W. (2015). The anterior insula shows heightened interictal intrinsic connectivity in migraine without aura. Neurology, 84(10), 1043–1050. 10.1212/WNL.0000000000001330 25663219 PMC4352101

[brb33316-bib-0037] Yeh, F. C. , Wedeen, V. J. , & Tseng, W. Y. (2010). Generalized q‐sampling imaging. Ieee Transactions on Medical Imaging, 29(9), 1626–1635. 10.1109/TMI.2010.2045126 20304721

